# Residual Dipolar Couplings for Resolving Cysteine Bridges in Disulfide-Rich Peptides

**DOI:** 10.3389/fchem.2019.00889

**Published:** 2020-01-22

**Authors:** Venkatraman Ramanujam, Yang Shen, Jinfa Ying, Mehdi Mobli

**Affiliations:** ^1^Centre for Advanced Imaging, The University of Queensland, St Lucia, QLD, Australia; ^2^Laboratory of Chemical Physics, National Institute of Diabetes and Digestive and Kidney Diseases, Bethesda, MD, United States

**Keywords:** disulfide-rich peptides, nuclear magnetic resonance, peptides, NMR, residual dipolar couplings, RDCs

## Abstract

Disulfide bridges in proteins are formed by the oxidation of pairs of cysteine residues. These cross-links play a critical role in stabilizing the 3D-structure of small disulfide rich polypeptides such as hormones and venom toxins. The arrangement of the multiple disulfide bonds directs the peptide fold into distinct structural motifs that have evolved for resistance against biochemical and physical insults. These structural scaffolds have, therefore, proven to be very attractive in bioengineering efforts to develop novel biologics with applications in health and agriculture. Structural characterization of small disulfide rich peptides (DRPs) presents unique challenges when using commonly applied biophysical methods. NMR is the most commonly used method for studying such molecules, where the relatively small size of these molecules results in highly precise structural ensembles defined by a large number of distance and dihedral angle restraints per amino acid. However, in NMR the sulfur atoms that are involved in three of the five dihedral angles in a disulfide bond cannot be readily measured. Given the central role of disulfide bonds in the structure of these molecules, it is unclear what the inherent resolution of such NMR structures is when using traditional NMR methods. Here, we use an extensive set of long-range residual dipolar couplings (RDCs) to assess the resolution of the NMR structure of a disulfide-rich peptide. We find that structures based primarily on NOEs, yield ensembles that are equivalent to a crystallographic resolution of 2-3 Å in resolution, and that incorporation of RDCs reduces this to ~1-1.5 Å resolution. At this resolution the sidechain of ordered amino acids can be defined accurately, allowing the geometry of the cysteine bridges to be better defined, and allowing for disulfide-bond connectivities to be determined with high confidence. The observed improvements in resolution when using RDCs is remarkable considering the small size of these peptides.

## Introduction

Disulfide bridges are naturally occurring cross-links formed between the side chains of two cysteine residues and are one of the most important post-translational modifications in proteins. The significance of disulfide bonds in proteins can be appreciated by their prevalence accounting for ~18% of all known protein structures (9,709 of the 55,032 proteins deposited in the PDB contain at least one disulfide bond – excluding structures with >90% identity, PDB accessed 2019-10-15).

Disulfide-rich peptides and proteins are commonly secreted, and include biopharmaceutical targets such as hormones and antibodies (Lewis and Garcia, 2003; Mamathambika and Bardwell, 2008; Gongora-Benitez et al., 2014). In these molecules, the disulfide bonds serve to stabilize the protein fold in the extracellular environment. This property is perhaps most dramatically demonstrated in venom peptides, where disulfide-rich peptide toxins are not only excreted but further injected into a foreign host where they exert their function, often with devastating consequences (Undheim et al., 2015).

The potency and portability of disulfide-rich peptides have attracted much attention from the growing biotechnology sector as a potential source of leads for development of therapeutics or agricultural agents (Gongora-Benitez et al., 2014). As research efforts in this field intensify there is an interest in defining the high-resolution structure of these proteins to interpret structure-activity relationship studies and ultimately for rational peptide engineering (Brust et al., 2013).

Structural analysis of small disulfide-rich peptides, however, presents unique challenges in commonly applied high-resolution biophysical characterization by NMR spectroscopy and X-ray crystallography. In X-ray studies the crystal packing forces can have a significant effect on the structure of these molecules (de Araujo et al., 2014), where the peptide fold is often more reliant on the disulfide bonds than an extensive hydrophobic core (Undheim et al., 2016). The dynamic nature of peptides, often including extended loops, can further complicate the crystallization process itself. An approach to overcome these problems is the use of racemic crystallization methods (Zawadzke and Berg, 1993), which have gained popularity with the reduced cost of production of the **D**-form of peptides (Yeates and Kent, 2012). Indeed, where sufficient quantities of the **D**-form of the peptide can be synthesized and folded readily, this provides an attractive approach to structural characterization of peptides.

**Figure 1:**
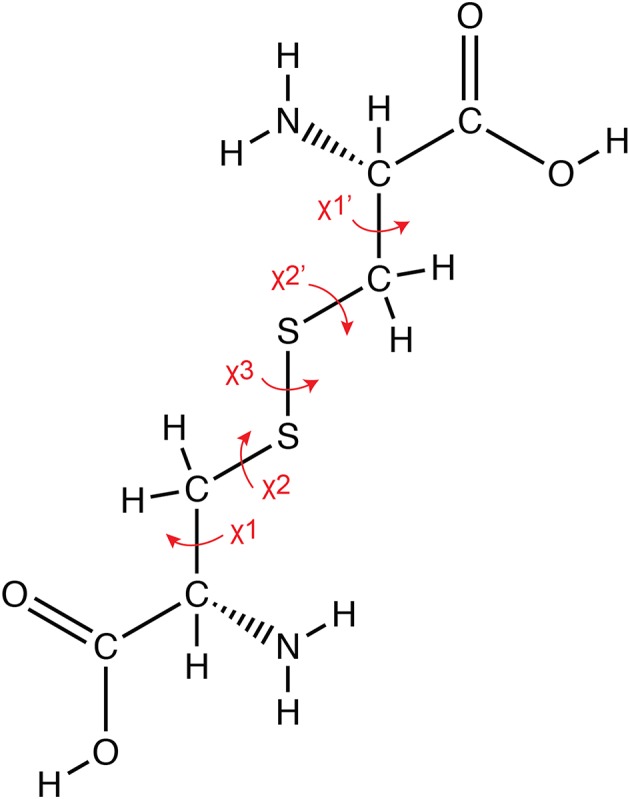
Geometry of a disulfide bond. The covalent bond between the sulfur atoms of cysteine residues results in the formation of a disulfide bond, which involves five sidechain torsion angles as indicated in the figure.

In NMR, the sulfur atoms that are involved in three of the five dihedral angles in a disulfide bond cannot be readily measured (Figure 1) (Mobli and King, 2010). This can be particularly detrimental to structure determination of these molecules, as the disulfide bonds may act as hinges, connecting distal parts of the peptide. Large inaccuracies in the definition of the geometry of disulfide bonds can therefore lead to reorientation of the relative position of different segments of the peptide. Efforts to replace sulfur with the NMR amenable ^77^Se isotope of selenium offer a solution (Mobli et al., 2009), and whilst very useful in defining disulfide connectivities, it remains unclear how the selenium itself affects the overall 3D structure.

Despite the above noted disadvantages, NMR remains the preferred method for structural characterization of peptides, with 73% (2524/3436) of all structures of peptides (proteins smaller than 6 kDa) solved by NMR spectroscopy. For disulfide containing peptides this statistic increases to 93% (800/853 – PDB accessed 2019-09, including only representative structures at >90% sequence identity). In principle, the small size of peptides makes them ideally suited to structural characterization by NMR spectroscopy, where the relatively small number of atoms provides largely unequivocal assignment of all atoms and their interatomic interactions. This is particularly true where uniform isotope labeling can be applied, allowing for use of multidimensional heteronuclear NMR methods (Ikura et al., 1990). Indeed, in general, a large number (>12) of restraints (distance and dihedral angle) per residue can be generated in these structures leading to a very high precision in the structure calculation step – RMSD of 0.1-0.2 Å often reported over ordered regions of the backbone (Klint et al., 2015; Undheim et al., 2015). However, the achieved precision can be deceptive as it reflects convergence of the numerical optimization and does not necessarily correlate with the accuracy of the structural models generated. Indeed, recent work investigating the structure of a series of disulfide containing proteins by NMR and X-ray crystallography found structural discrepancies (0.5–0.8 Å along the C_α_ atoms) (Alex et al., 2019), with the NMR structures having higher calculated disulfide bond energies (Schmidt et al., 2006).

Residual dipolar couplings (RDC) provide an excellent independent measure of structural accuracy of NMR models and can themselves be used to improve the resolution of NMR structures (Tjandra and Bax, 1997; Bax and Grishaev, 2005). Here, we seek to apply RDCs in assessment of the accuracy of disulfide-rich peptide structures generated by NMR spectroscopy and also investigate if these structures can be further refined by the inclusion of RDCs in the refinement step. We perform an in-depth structural analysis of a disulfide-rich peptide (Ta1a) previously reported with a precision below 1 Å using standard heteronuclear NMR methods (Undheim et al., 2015). Our results show that the accuracy of this structure is consistent with an X-ray structure of ~2.5 Å resolution. RDC refinement improves this to the equivalent of a 1-1.5 Å X-ray structure resolution. At this resolution there is a significantly better definition of sidechain orientations, and critically, improved definition of the cysteine bridges and their connectivities.

## Materials and Methods

### Ta1a Production

A pLicC vector harboring a codon optimized Ta1a gene (GeneArt, Life technologies) was transformed into BL21 (*DE3*) *E. coli* cells. A single colony was used to inoculate a culture and grown over night in 100 ml Luria-Bertani (LB) media containing 100 μg/ml ampicillin and the culture was grown at 37°C at 200 rpm until the optical density at 600 nm (OD_600_) reached 0.8. 5% inoculum was used from the starter culture to inoculate 1 L of LB medium containing 100 μg/ml of ampicillin.

The culture was induced at an OD_600_ of 0.8, with IPTG (isopropyl-β-D-thiogalactopyranoside) at a final concentration of 500 μM, and then further grown for another 14 h at 18°C. The bacterial cells were harvested by centrifugation at 6,000 rpm for 20 min at 4°C, and then resuspended in 10 ml of lysis buffer (40 mM Tris, 300 mM NaCl, 10 mM imidazole pH 8.0). The cells, kept on ice, were then lysed using sonication. Subsequently, the supernatant was collected after centrifugation at 17,000 rpm for 45 min at 4°C and filtered through a 0.45 μm filter.

The cell lysate was applied to a buffer-equilibrated, 5 ml His-Trap column (GE Healthcare) using a peristaltic pump at a flow rate of 3 ml/min. The column was then washed with 30 column volumes of wash buffer (40 mM Tris, 300 mM NaCl, 40 mM imidazole pH 8.0). The protein was eluted with 40 mM Tris, 300 mM NaCl at pH 8.0 with 250 mM imidazole. The eluted protein was concentrated and buffer exchanged using 15 ml centrifugal filters (Millipore) with a 10 kDa cut-off membrane, using a Tris buffer (40 mM Tris, 300 mM NaCl, pH 8.0) to remove imidazole.

Ta1a was separated from the (His)_6_-MBP fusion by Tobacco Etch Virus (TEV) protease. The cleavage was performed by adding TEV protease (1 mg/ml) to the protein solution [at a UV absorption at 280 nm (A_280_) ratio of 1:20] in a redox buffer (2.5 mM GSH and 0.25 mM GSSG) and incubated at 25°C overnight. The reaction mixture was loaded onto a 5 ml His-Trap column (GE Healthcare) and the flow-through containing Ta1a was collected.

The Ta1a sample was acidified with 0.05% Trifluoroaceticacid (TFA) and filtered through a 0.45 μm filter, and loaded onto a semi-preparative column (C3-Zorbax resin, Agilent) at a flowrate of 3 ml/min with a linear gradient of 5-80% acetonitrile (0.043% TFA) in water (0.05% TFA) over 50 min using an Agilent HPLC system. Elution was monitored by UV absorption at 214 and 280 nm. The fraction containing the pure peptide was lyophilized and stored at −20°C.

Uniformly enriched protein was produced by growing the transformed *E. coli* cells in minimal media supplemented with 4.0 g/L ^13^C_6_-glucose and 1.0 g/L ^15^NH_4_Cl as the sole carbon and nitrogen sources, respectively (Marley et al., 2001).

### Preparation of Liquid Crystalline Solutions

A Pf1-phage aligned sample was obtained by mixing the stock solution of 50 mg/ml Pf1 phage (http://www.asla-biotech.com) with the protein solution and gently pipetting the final mixture a few times. PEG solution (Ruckert and Otting, 2000) was prepared by mixing the pentaethylene glycol monododecyl ether (C12E5; Sigma Aldrich), with hexanol at a molar ratio [PEG]:[hexanol] of 3:2. All the anisotropic data in aligned media were recorded at 25°C.

### NMR Measurements

Details of all NMR experiments are provided in Tables S1, S2. First, a 3D CT-HNCA (Grzesiek and Bax, 1992) spectrum was recorded to confirm the assignments of Ta1a in 20 mM phosphate buffer pH 6.2 against the published values (BMRBID: 16667). All subsequent experiments were performed in the same buffer using a peptide concentration of ~500 μM (unless otherwise stated).

For RDC measurements data were acquired under isotropic conditions as well as when using the two different alignment media. Where values deviate from the details in Table S2, these are provided here. 2D IPAP-HSQC (Ottiger et al., 1998) spectra were obtained by acquiring two datasets in an interleaved manner for measurement of ^1^*J*_NH_ splittings. 3D CT-HN(CO)CA (Bax et al., 2001) spectra without H_α_ decoupling were recorded for measurement of ^1^*J*_Cα*Hα*_. 3D HNCO (Bax et al., 2001) spectra without C_α_ decoupling were acquired for measurement of ^1^$J_{{\text{C}}^{\prime }C\alpha }$ splittings. In this case, for the isotropic sample, the data was acquired using non-uniform sampling mode and the data reconstructed using the sparse multidimensional iterative lineshape-enhanced method (Ying et al., 2017). 3D CT-HN(COCA)CB (Li et al., 2015) spectra without H_β_ decoupling were recorded for measurement of sums of ^1^*J*_Cβ*Hβ*2_ and ^1^*J*_Cβ*Hβ*3_ splittings.

For χ_1_ measurements a 3D HA[HB,HN](CACO)NH (Lohr et al., 1999) spectrum was acquired to obtain ^3^*J*_Hα*Hβ*_ couplings. A 3D HNHB (Archer et al., 1991) spectrum was recorded for the measurement of ^3^*J*_NHβ_ coupling constants.

A 3D ^13^C-edited NOESY (Davis et al., 1992) spectrum as well as a 3D ^15^N-edited NOESY (Kay et al., 1992) spectrum, each using a mixing time of 150 ms, were recorded at 900.1 MHz ^1^H frequency for stereospecific assignment of H_β2_ and H_β3_ protons from H_α_-H_β2_, H_α_-H_β3_, H_N_-H_β2_, and H_N_-H_β3_ cross-peak intensities. Steady-state ^1^H-^15^N heteronuclear NOEs were recorded at 900.1 MHz ^1^H frequency as a qualitative probe for large amplitude backbone dynamics.

The NMRPipe software system (Delaglio et al., 1995) was used for processing the 3D CT-HNCA, 2D IPAP-HSQC, 3D HNCO, 3D HN(CO)CA, 3D CT-HN(COCA)CB and 3D HA[HB,HN](CACO)NH spectra. The 3D HNHB, 3D ^13^C edited NOESY, ^1^H-^15^N heteronuclear NOE and 3D ^15^N edited NOESY data were processed using the Rowland NMR toolkit (Hoch and Stern, 1996). The CCPNMR (Vranken et al., 2005) and Sparky (Goddard and Kneller, 2008) programs were used for analysis. Peak positions and intensities were determined using parabolic interpolation in all three dimensions of local peak maxima. Resonance assignments of Ta1a were made using the acquired spectra in agreement with previously published data (Undheim et al., 2015).

### Refinement of the Ta1a Structure

The structure of Ta1a was refined starting from the coordinates of the PDB deposition 2KSL (Undheim et al., 2015), against the N-H_N_, C_α_-H_α_, C′-C_α_, N-C′ and ΣC_β_H_β_ RDCs (in both alignment media where available) using the program XPLOR-NIH (Schwieters et al., 2003), which uses a simulated annealing protocol. The RDC refinements are here performed using a standard Cartesian molecular dynamics simulated annealing refinement protocol, starting from the coordinates of 2KSL structure, and with all structural restraints used in the CYANA calculations (Table S7). The protocol included 200,000 steps of 1 fs each, with the temperature linearly ramped down from 1000 to 25 K, followed by a Powell energy minimization. Empirical force fields included quadratic bond, angle, and improper terms with force constants of 5,000 kcal Å^−2^ mol, 500 kcal rad^−2^ mol^−1^ and 500 kcal rad^−2^ mol, respectively, as well as a quartic repulsive-only non-bonded potential with a force constant of 4 kcal Å^−2^ mol^−1^. In addition, backbone/backbone hydrogen bonding geometries were restrained via a potential of mean force (HBDB term in XPLOR-NIH). Varied magnitude alignment tensors were used for the RDCs of each alignment condition during the structural calculations. Force constants for different types of RDCs in two different alignment media were obtained from a combination of force constants (0.20, 0.15, 0.20, 0.20, 0.15 kcal Hz^−2^ mol^−1^for ^1^*D*_NH_, ^1^*D*_CaHa_, ^1^$D_{{\text{C}}^{\prime }\text{N}}$, ^1^$D_{\text{Ca}{\text{C}}^{\prime }}$ and ^1^*D*_CbHb_, respectively, from Pf1 phage medium; 0.20, 0.20, 0.10, 0.20 kcal Hz^−2^ mol^−1^ for ^1^*D*_NH_, ^1^*D*_CaHa_, ^1^$D_{{\text{C}}^{\prime }\text{N},}$ and ^1^$D_{\text{Ca}{\text{C}}^{\prime }}$, respectively, from PEG liquid crystals medium, and with all the values being normalized^7^ to the ^1^*D*_NH_ couplings) that yielded the best cross validation performance according to a grid searching procedure. The ^1^*D*_NH_ RDC force constant multipliers (and thereby the multipliers for the other types of RDCs) were ramped up with a constant multiplicative factor throughout the protocol from 0.05 to 5.0; i.e., at 25 K, the ^1^*D*_NH_ force constant was ramped up to 1 kcal Hz^−2^ mol^−1^. A total of 50 structures was generated, and the twenty lowest energy structures were retained and then deposited in the PDB (entry 6URP). All figures of protein structures were prepared using PyMol (Schrodinger, 2015).

## Results

### Protein Expression and Purification

We transformed a plasmid containing the gene encoding a Ta1a-fusion protein into *E. coli* (BL21*(DE3)* strain) cells for expression. This gene also includes a periplasmic localization sequence followed by a (His)_6_ tagged maltose binding protein (MBP) – both N-terminal to the peptide sequence. The fusion also includes a TEV-protease cleavage site between the peptide and the fusion partner. Using this construct, we purified the fusion protein using IMAC chromatography followed by cleavage of the peptide from the fusion partner by TEV protease. The TEV protease and the released fusion partner were removed by an additional round of IMAC chromatography. We further purified the peptide using reverse-phase HPLC (Figure S1). The final yield of Ta1a was ~0.6 mg/L.

### Optimization of Alignment Media Concentrations for RDC Measurements

We used two liquid crystalline media that align the protein differently relative to the magnetic field: a suspension of the negatively charged filamentous phage Pf1 (Hansen et al., 1998) and a polyethylene glycol (PEG) based liquid crystal (Ruckert and Otting, 2000). The prepared liquid crystalline medium will not always align in the magnetic field, if it does, the protein will also align. There is also a probability the protein will interact with the alignment media resulting in a higher degree of alignment than desired. Hence the strength of alignment in the particular liquid crystalline medium needs to be assessed. To determine the level of alignment of the liquid crystals themselves and how the peptide aligns with the Pf1 medium, we acquired a series of ^2^H spectra and ^1^H spectra of Ta1a, while reducing the Pf1-phage concentration from a starting value of 20 mg/ml. We found that the highest concentration of Pf1 phage at which the ^1^H spectrum shows good agreement with its isotropic counterpart (by visual comparison) is 5.8 mg/ml of Pf1 phage (Figures S2, S3). Similarly, we optimized the PEG bicelles concentration by measuring RDC data in either 5 or 8% w/v of PEG (Figure S4). Both concentrations yield good spectral data, with good agreement of backbone amide residual dipolar couplings (^1^D_NH_) when compared to the back-calculated values from the published Ta1a structure (2KSL). Based on the magnitudes of ^1^H-^15^N couplings, we chose the higher PEG concentration as it resulted in RDCs having a favorable magnitude in the 15-20 Hz range.

We used the optimized alignment conditions to acquire NMR data for subsequent structural refinement. The NMR data included a number of two-dimensional (2D) and three-dimensional (3D) experiments (Table S1), for extraction of *J*-splittings used to derive RDCs and dihedral angles. The majority of experiments were acquired under isotropic and two different anisotropic conditions, resulting in a total of 16 datasets.

### J-Coupling Measurements and Analysis of χ_1_ Angles and Rotameric Distribution

To improve the resolution of the peptide sidechains, we analyzed the χ_1_ rotamer positions and distributions using a combination of *J*-couplings, NOE intensities and RDCs.

First, we assigned prochiral β-methylene protons using a combination of ^3^*J*_Hα*Hβ*_ and qualitative ^3^*J*_N−Hβ_ couplings (Bax et al., 1994). These coupling constants have a characteristic pattern for each of the three energetically preferred staggered rotamer positions (χ_1_ = 60°, 180° or −60°) (West and Smith, 1998). Following this procedure, we were able to stereospecifically assign 16 β-methylenes in Ta1a (Table S3). For 15 residues we found evidence of motional averaging, with ^3^*J*_Hα*Hβ*_ couplings in the range of 5.0-9.0 Hz and/or qualitative ^3^*J*_NHβ_ couplings classified as “medium-medium” pairs. In these cases, the χ_1_ angle was classified as “average.” We were unable to determine the χ_1_ angle of six residues due to overlap of their H_β2_ and H_β3_ resonances.

For valine, isoleucine and threonine residues (each with a β-methine proton), the side-chain is assumed to adopt a single staggered conformation when the measured ^3^*J*_Hα*Hβ*_ couplings is greater than ~10 Hz (~9 Hz for Thr because of the effects of high electronegativity of the oxygen substituent) or less than ~5 Hz (Smith et al., 1991; Li et al., 2015). Three out of the 6 residues (Table S4) in Ta1a fit into this category with supporting data from the qualitative ^3^*J*_N−Hβ_ measurements. For cases where the ^3^*J*_Hα*Hβ*_ couplings does not fit into this category, the side-chain may either occupy a non-staggered conformation or be a rapid average of multiple conformations. Solvent exposed residues Ile-6 and Thr-12 exhibit this behavior, likely as a consequence of conformational averaging.

χ_1_ rotamer positions can also be derived from characteristic intra-residue ^1^H-^1^H NOE intensities. We were, therefore, able to examine the consistency of the above determined χ_1_ angles with experimentally measured NOE intensities (Table S5). Overall, we found good agreement between the two datasets, however, for Glu-36, Phe-38 and Asp-41, there is an apparent inconsistency. For these residues the H_N_-H_β2_ and H_N_-H_β3_ NOE intensities suggest χ_1_ rotamer averaging whereas the analysis of the *J*-couplings is consistent with a single staggered rotamer position. Given the apparent uncertainty, the χ_1_ angles determined for these residues were excluded from the subsequent structural refinement step.

Finally, we can also determine the χ_1_ rotameric states by comparison of appropriately scaled pairs of RDCs (Chou and Bax, 2001). This is done by assuming that the C_β_–H_β_ bonds are at staggered conformations, and parallel to the C_α_–H_α_ and C_α_–C′ bonds. Under these conditions the sum of D_Cβ*Hβ*2_ and D_Cβ*Hβ*3_ is related to normalized sum of either:

[1] (C_α_–H_α_)_i_ and (C_α_–C′)_i_, or
[2] (C_α_–H_α_)_i_ and (C_α_–C′)_i−1_ of the preceding residue, or
[3] (C_α_–C′)_i_ and (C_α_–C′)_i−1_ of the preceding residue.

Close agreement with [1], [2] or [3] indicates a χ_1_ value of 180°, 60°, or −60°, respectively (Table S6 shows examples of this analysis). For example, in Phe–16, Cys–26, Tyr–43, and His–47 the D_Cβ*Hβ*2_+D_Cβ*Hβ*3_ is closest to the sum of ${\text{D}}_{{\text{C}}^{\prime }C\alpha }$ and D_Cα*Hα*_, indicating a χ_1_ angle of 180°. For Asn-40 the sum of D_Cβ*Hβ*2_ and D_Cβ*Hβ*3_ is closest to the sum of intraresidue D_Cα*Hα*_ and ${\text{D}}_{{\text{C}}^{\prime }C\alpha }$ of the preceding residue, indicating a χ_1_ angle of 60°. This analysis does not clearly identify residues that exhibit χ_1_ = −60°, because in these cases the intra-residue (C_α_–N)_i_ vector is not exactly parallel to the preceding residue's (C_α_–C′) bond and therefore additional data is required to unambiguously identify the χ_1_ as being either −60° or 180°. Nevertheless, this approach is useful for extracting χ_1_ rotamer information for a significant fraction of residues in a protein, and further complements the conventional rotamer analysis using *J*-couplings and NOE data.

### Structural Refinement of the Ta1a Structure

We wanted to compare the previously published structure of Ta1a with that of the refined structure using the RDCs and dihedral angles determined here. The previously reported structure (Undheim et al., 2015) of Ta1a was calculated using NOE-based distance restraints supplemented with backbone dihedral-angle restraints derived from TALOS chemical shift analysis and hydrogen bonding restraints (Cornilescu et al., 1999). This data was further supplemented with stereospecific assignments and χ_1_ angles obtained from initial structure calculations.

Compared to the published structure we have made a number of changes in the CYANA structure calculation protocol. First, to identify disordered residues, we acquired a heteronuclear NOE dataset, where ^15^N-^1^H NOEs are used as reporters of fast dynamics along the peptide backbone. Overall, the structure was found to be highly ordered except for residues E2, I6, and K51 (Figure S5). RDC and dihedral angle restraints involving these residues were thus removed in all subsequent structure calculation steps.

Next, we replaced the computationally derived stereospecific assignments and χ_1_ angles with those we have determined experimentally in this study. In our initial CYANA calculations, using this new data, we found that inclusion of the χ_1_ angle of N41 led to Ramachandran violations. We note that the sidechain of this residue also shows evidence of hydrogen bonding in the D_2_O exchange experiment, although we were here not able to unambiguously determine the hydrogen bonding partner of this residue. Both the χ_1_ and sidechain hydrogen bond constraints involving this residue were excluded from subsequent analyses. All other hydrogen bonds based on D_2_O exchange were included as previously assigned. No further violations were observed in subsequent CYANA calculations using these updated parameters.

The RDC refinements are here performed using NIH-XPLOR, thus the CYANA constraints were translated to the appropriate format for this software. In NIH-XPLOR, all constraints are weighted equally (1.0), and initial structure calculations revealed that some of the experimentally determined dihedral angles were being violated in some structures. Thus, to reflect the higher confidence of the experimentally derived dihedral angle constraints, we used a higher weighting for these (3.0 vs. 1.0), which resolved the observed violations without introducing any additional ones. These constraints formed the basis of all subsequent structure calculations (with and without inclusion of RDCs).

### Measurement of Backbone and Side-Chain RDCs

RDCs can be used to both assess the resolution of an existing structure and to improve the resolution of a structure during the refinement step. To assess the resolution of the published Ta1a structure we fitted the backbone RDCs to the existing structure by order matrix analysis using singular value decomposition (SVD) (Losonczi et al., 1999). This method returns the predicted RDCs and the parameters of the alignment tensor determined by the fitting procedure. To quantify the agreement between the structure and the measured dipolar couplings, the quality factor Q is used as proposed by Cornilescu et al. (1998):

(1) $$\begin{array}{l} Q=\frac{\text{rms}\left({\text{D}}_{\text{calc}}-{\text{D}}_{\text{obs}}\right)}{\text{rms}\left({\text{D}}_{\text{obs}}\right)} \end{array}$$

where D_calc_ and D_obs_ are calculated and observed dipolar couplings in the above equation (Equation 1). This factor offers a straightforward and unambiguous way to evaluate the structural quality.

**Figure 2:**
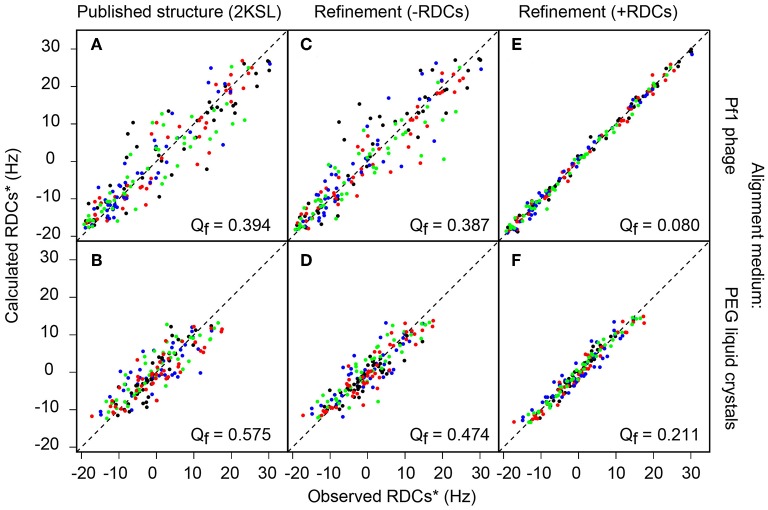
Plots of experimental vs. back-calculated RDCs using different Ta1a structures. The three columns refer to different structures, the left column **(A,B)** show data for the published structure, the middle column **(C,D)** show data for the structure refined here without RDCs and the right column **(E,F)** show data for the structure refined here using RDCs. The two rows show data using different alignment media (Pf1 phage top and PEG liquid crystals bottom). All RDCs (^1^D_Cα*Hα*_ in green, ^1^${\text{D}}_{\text{Cα}-C^{\prime }}$ in red, ^1^${\text{D}}_{\text{N}-{\text{C}}^{\prime }}$ in blue) are scaled with respect to ^1^D_NH_ (in black) RDCs.

The Q factor obtained for the published Ta1a structure was compared with the RDC data from the Pf1 phage and PEG aligned samples, where Q factors of 0.39 and 0.58 were found, respectively (Figure 2). In literature reports, Q factors of ~0.4 are commonly found for structures with a resolution equivalent to an X-ray crystallographic resolution of 2–3 Å resolution (Chen and Tjandra, 2011).

To refine the structure of Ta1a, we used all of the RDC data generated from the experiments listed in Table S1. This included a total of 355 backbone RDCs and 37 sidechain RDCs. The sidechain RDCs were derived from the sum of ^1^H_β_-^13^C_β_ RDCs in the 3D HN(COCA)CB (Li et al., 2015) spectrum recorded without ^1^H decoupling in the ^13^C_β_ dimension in the Pf1 phage aligned protein sample. In this experiment we were able to define side-chain RDCs for 37 out of the 49 non-Gly residues. Inclusion of these RDC restraints resulted in a computed structural ensemble having a Q factor of 0.08 and 0.21 in the Pf1 phage and PEG aligned samples, respectively (Figure 2 and Table S7).

Since the RDC data used to derive the structure is also used to calculate the Q factor, there is a clear potential for overfitting, and an alternative approach is required to assess the resolution of our RDC refined structure. This can be achieved by omitting some of the RDCs, and to use these excluded RDCs to cross-validate the refined structure. This procedure allows for an unbiased “Q_free_-value” to be determined. Here, we omitted 10% of all RDCs in each of the eight experiments (N-H_N_, C_α_-C′, C_α_-H_α_and C′-N in two alignment media) involving backbone atoms. The refinement was repeated using this reduced dataset, and the back-calculated values of the omitted RDCs were used to calculate the Q_free_ value. The procedure was repeated 10 times (ensuring each RDC was left out across the 10 runs) and the average Q_free_ value was 0.12 ± 0.03 for the Pf1-aligned sample and 0.24 ± 0.02 for the PEG-aligned data. A Q_free_ value in the low 20% range (0.20) roughly translates to structures consistent with an X-ray crystallographic resolution of 1–1.5 Å resolution.

## Discussion

We have used residual dipolar couplings (RDCs) to assess the resolution of a disulfide-rich peptide structure. This structure had previously been solved using a large number of NMR restraints derived from high-quality heteronuclear NMR data (Undheim et al., 2015). Although the structure of this relatively small peptide had been defined with high precision, the quality of the structure is similar to what is typically achieved using similar methods for larger proteins (~2−3 Å). We subsequently used the measured RDCs to see if we could improve the resolution of this structure further. Our results show that inclusion of RDCs dramatically improves the attainable resolution.

### Resolution of Peptide Structures

NMR remains the preferred method for solving peptide structures. Analysis of the protein databank (2019/10) reveals that three quarters of PDB structures of peptides smaller than 6 kDa have been solved by NMR spectroscopy. The fast-molecular tumbling of these molecules results in sharp NMR lines and the relatively low number resonances further reduces the complexity of the spectral data.

The favorable NMR conditions experienced by peptides in solution, results in data with low levels of ambiguity and in principle in a better-defined structure. It is, therefore, not unusual to find peptide structures that are defined by more than 10 experimental restraints per amino acid, yielding structural ensembles computed with a precision of 0.1–0.5 Å root-mean-squared difference (RMSD) over structured regions, along the peptide backbone (de Araujo et al., 2014; Klint et al., 2015; Undheim et al., 2015).

It remains unclear, however, if the high precision of peptide structural ensembles reflects the accuracy of these structures. The high precision is a direct consequence of the level of ambiguity in the data, which has been argued to be a good corollary with the accuracy of an NMR structure (Tikole et al., 2013; Buchner and Guntert, 2015). Thus, whilst it would appear reasonable to assume that the higher precision achieved for small peptides makes these more accurately defined, we note that there is no consensus on how NMR accuracy should be defined (Rosato et al., 2013).

In the case of disulfide rich peptides, there is a unique challenge arising from NMR blind-spots near the sulfur atoms. This arises as NMR signals from sulfur atoms cannot be readily measured in macromolecules. In the case of methionines this is not a significant concern as inaccuracies in defining the local environment about the sulfur atom only results in lower accuracy of side-chain dihedral angles near the periphery of the amino acid. In disulfide bonds, however, the quiescent sulfur atoms obscure three of the five dihedral angles that connect backbone atoms of often distal segments of the peptide.

The non-uniform distribution of structural restraints in disulfide-rich peptides has the potential of providing deceptively favorable ensemble statistics—in particular for helical peptides. In general NMR structures are defined overwhelmingly by short range NOE interactions within an amino acid or between neighboring amino acids. Orientation of segments of secondary structure are, however, often organized either through backbone-to-backbone hydrogen bonds (in β-sheets) or in proton-rich regions in the hydrophobic core of the protein. Thus, helices or loop regions that are connected by disulfide bonds in peptides rely critically on well-defined disulfide bonds.

In this study we revisited the high-precision structure of Ta1a, a largely helical disulfide-rich peptide. The peptide contains a disulfide bond connecting two helices as well as two disulfide bonds connecting a loop region with a helix. The peptide structure was solved using a large number of NOE and dihedral angle restraints generated by heteronuclear NMR measurements using an isotope-labeled sample. The peptide displays excellent NMR properties and consequently the structural ensemble can be computed with very high precision.

**Figure 3:**
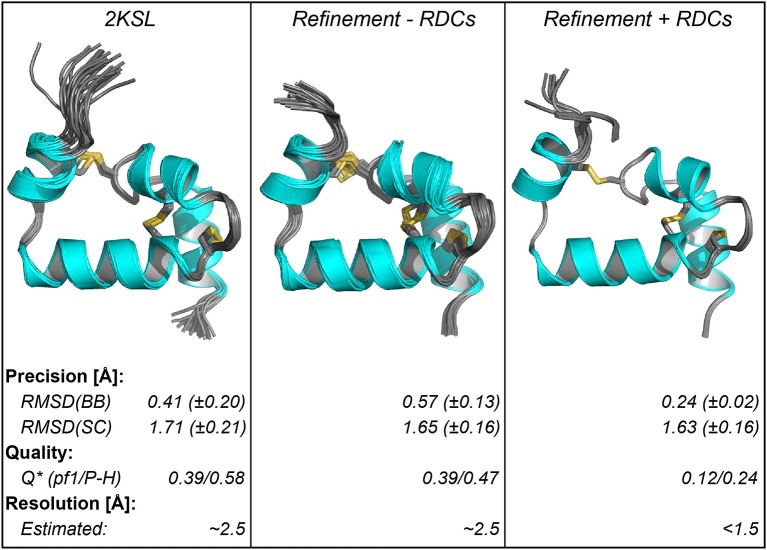
Structural parameters of ensembles of Ta1a structures using different refinement methods. The left panel shows the published structure, the middle and right panels show the refinement of the structure based on additional data acquired here, either excluding the RDC data (middle) or including the RDC data (right). Below the structure the structural parameters are summarized. The quality factor (Q) was calculated using the RDC data in the two different alignment media (Pf1-phage [pf1] and PEG-hexanol liquid crystals [P-H]). The data show that improvement in Q factor when including RDCs is consistent with a crystallographic resolution where sidechains can be resolved. ^*^The quality factor for the RDC refined structure was determined using a procedure where RDCs used in the refinement are excluded when deriving the Q factor (Q_free_).

We used RDCs to assess the accuracy of the Ta1a structure and found it to be substantially lower than the reported precision. The Ta1a structure was originally reported with a precision of ~0.4 Å along the structured regions of the backbone. In this study we have refined the original structure using additional J-coupling and 3D NOE-derived restraints and an additional molecular dynamics refinement step in Xplor-NIH (Schwieters et al., 2018). The precision is still very high, albeit slightly lower than the reported structure (0.6 Å along the structured regions of the backbone). We then assessed the quality of the structure using an extensive RDC dataset acquired in two different alignment media (392 RDCs in total with 355 backbone RDCs). The agreement of the structures with the measured RDCs reveals that the two structures have similar quality factors, with the structure refined here agreeing slightly better than the published structure with the RDC data measured in the PEG-hexanol liquid crystals (see Figures 2, 3). The quality factors themselves are consistent with structures that have an equivalent crystallographic resolution of ~2.5 Å (Bax, 2003). Indeed, when we align the two structures along their structured regions there is a ~1 Å RMS difference in atomic coordinates (over residues 3–5 and 7–50).

We next used the RDC data to refine the peptide structure, which resulted in a structure that fits the RDC data very well (Figure 2). It is important to validate these results by omitting some of the measured RDCs to see how these fit the calculated structure using the remaining RDCs. Given the large number of RDCs generated here we excluded 10% of the backbone RDCs in each dataset (~35 RDCs randomly omitted from ~350) and performed the structure calculation using the remaining constraints. We repeated this procedure ten times, each time randomly omitting a different set of 10%. The quality factor generated using the omitted RDCs shows that the structure is of high quality consistent with a high-resolution crystal structure (1-1.5 Å) (Bax, 2003). We also aligned the ten generated structures with each other and to the structure generated using all RDCs. In each case the RMSD between these structures was <0.2 Å (all atoms in structured regions: residues 3-5 and 7-50).

**Figure 4:**
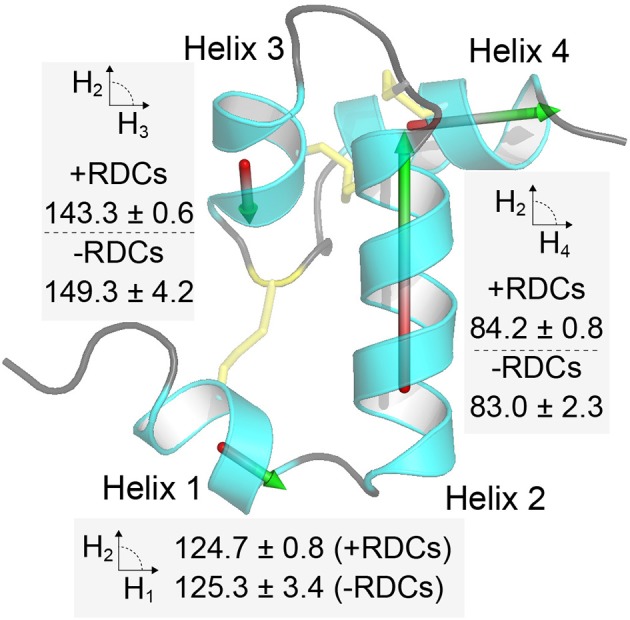
Inter-helix orientations are better defined when including RDC restraints. In the figure each of the four helices in Ta1a is represented by a vector, and in each case the angle between each vector and the central helix 2 vector is calculated. The procedure is repeated for each structure in the ensemble and the average and standard deviation provided in the figure near the relevant helix. The procedure applied to the ensemble of structures when RDCs are included or omitted in the structure calculation step (+ and – RDCs, respectively). Helix 3 shows the largest deviation and spread of orientations when RDCs are not included.

Having the three structures (published, refined here with and without RDCs – Figure 3) we investigated what the likely source of discrepancy between them was. Given that there was a ~1 Å difference in structural alignment between the two structures that were generated without RDCs, when we align each of these with that solved using the RDCs. We found that both of these were ~1 Å different to the RDC refined structure as well (2KSL = 1.2 Å, Xplor-NIH refinement without RDCs = 1.0 Å). When we compared the alignment of each individual helix from either the published structure or that refined here without RDCs we find these to align very well with the RDC refined structure along the backbone (RMSD ~ 0.2 Å), indicating that the helices are locally accurately defined in all structures. The difference is, therefore, likely to be in the alignment of the helices with respect to each other. To test this, we represented each helix with a vector and calculated the angle formed between the central Helix-2 vector and the remaining three vectors (Figure 4). We find that Helix-3 is particularly displaced with respect to Helix-2. Further we find that when RDCs are not used in the refinement there is a much larger spread of inter-helix vector angles between different members of the same ensemble. This results in a larger standard deviation of the average angle within each structural ensemble (see Figure 4). The difference in average angle between Helix-2 and Helix-3 may appear to be small, but a 6° displacement of two connected 10 Å vectors is equivalent to about a 1 Å rotation at the tip of one the vectors. Thus, the vectorial displacement of the helical elements in a structure may be a better indicator of the resolution of a helical peptide than the ensemble RMSD.

### Defining Disulfide Geometries in Peptide Structures

Conformations of disulfide bridges are classified based on the five side-chain dihedral angles as shown in Figure 1. Different methods have been proposed to classify disulfide conformers (Srinivasan et al., 1990; Harrison and Sternberg, 1996; Hutchinson and Thornton, 1996; Schmidt et al., 2006; Ozhogina and Bominaar, 2009). Here, we used the method proposed by Schmidt et al. (2006). There are three basic disulfide types based on the combination of signs of the χ_2_, χ_3_, and χ_2_' angles and they are designated spirals, hooks or staples. The classification depends on the sign and order of the angles, for instance all positive or all negative angles are designated as spirals. Disulfide bonds are further classified as right handed (RH) or left handed (LH) depending on whether the sign of the χ_3_ angle is positive or negative, respectively. Schmidt et al. included the χ_1_ and χ_1_′ angles to further refine the classification (Schmidt et al., 2006). This has expanded the number of types from 6 to 20 different types.

**Figure 5:**
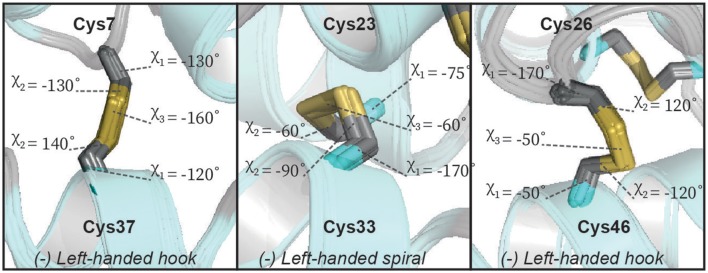
The geometry of the disulfide bonds of Ta1a. Ta1a contains three disulfide bonds, each panel shows expanded regions of the structure focusing on one of these disulfide bonds. The relevant sidechain dihedral angles defining the disulfide bond geometry are shown (χ_1_, χ_2_, and χ_3_). Two of the disulfide bonds form a left-handed hook while the central disulfide bond forms a left-handed spiral.

Using the above classification system, we analyzed the geometry of the three disulfide bonds in our Ta1a structures. In the NOE-derived Ta1a structure, the Cys_7_–Cys_37_ disulfide bridge exhibits 3 different conformers (–RH-hook; –RH-staple; –LH-spiral), the Cys_23_–Cys_33_ disulfide bridge predominantly adopts a –LH-spiral conformation with 9 structural models also adopting the +/−LH-spiral and for the Cys_26_–Cys_46_ disulfide bridge all the structural models in the ensemble adopt the +/−RH spiral conformation (Table S8)—note that the sign refers to the sign of the χ_1_ and χ_1_' angles. After refining the NOE derived structure with RDC restraints, the ensembles of all 20 structural models uniquely adopt –LH-hook, –LH-spiral, and –LH-hook for the disulfides Cys_7_–Cys_37_, Cys_23_–Cys_33_, and Cys_26_–Cys_46_, respectively (see Figure 5). We also compared our findings with those obtained using predictions from the DISH software (Armstrong et al., 2018). This software uses a trained neural network to predict the rotameric state of χ_1_ and χ_2_ dihedral angles (assuming idealized geometries) in disulfide bonds from input chemical shift values. The software produced reliable angles (>90% probability) for χ_2_ of residues 23 (180°), 33 (−60°) and 37 (180°). Compared to our RDC refined structure, the algorithm correctly predicted the rotameric state of residues 33 and 37, while residue 23 deviates from our results. The lack of reliable predictions for the other χ angles and the observed discrepancy may reflect structural heterogeneity as discussed further below.

**Figure 6:**
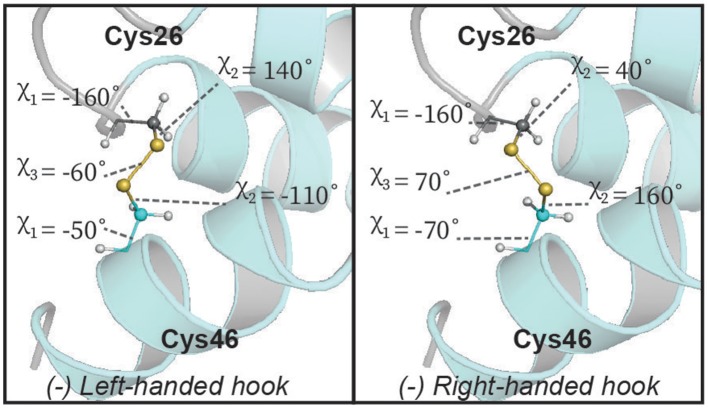
Alternative geometry of the central disulfide bond in Ta1a. The lowest energy structures describing Ta1a all have the same (left-handed hook) conformation. However, some of the higher energy solutions display a right-handed hook conformation which does not violate the dihedral angle restraints used (χ_1_). This alternative orientation also retains all C-H and C-C vectors in the disulfide bond in similar positions, suggesting that this type of heterogeneity is unlikely to be resolved by currently available NMR measurements.

Although we are able to classify the geometry of our disulfide bonds qualitatively, we note that there are some notable deviations from idealized geometries. Energetically the χ_1_ and χ_2_ angles in disulfide bonds have minima between −30° and −90° (gauche^−^), 30° and 90° (gauche^+^) and between 150° and −150° (trans). The χ_3_ angle has minima between −60° and −120° (left) as well as between 60° and 120° (right). The disulfide between Cys_23_–Cys_33_ fits into these limits whereas the disulfides between Cys_7_–Cys_37_ and Cys_26_–Cys_46_ do not satisfy the defined limits of the χ_1_/χ_2_ and χ_2_ angles, respectively (Figure 5). The χ_1_ rotamer analysis from *J*-couplings and NOE data further supports that Cys_7_ and Cys_37_ exhibit rotameric averaging. As χ_1_ is not locked in a staggered rotamer position this will affects the degree of freedom of the χ_2_ angle thereby exceeding the defined limits. While Cys_7_–Cys_37_ shows averaging at the level of the χ_1_ angle, Cys_26_–Cys_46_ shows a well-defined χ_1_ dihedral angle, but we find non-ideal χ_2_ angles. Further investigation of this disulfide bond revealed that some of the higher energy structures generated during structure calculations had a slightly different configuration of this bond (Figure 6). In the two alternative structures, we find a flip of the handedness of the disulfide bridge. What is particularly interesting is that while the χ_2_ and χ_3_ angles vary in these structures the χ_1_ angles remain largely the same (close to idealized staggered positions). Furthermore, the relative orientation of the C-H and C-C bond vectors remain largely the same, suggesting that the RDC restraints in this case would not be able to easily resolve this problem. This observed heterogeneity highlights the challenge in defining χ_2_ and χ_3_ angles by NMR spectroscopy—and suggests that beyond defining the χ_1_ angle we are largely reliant on the internal forcefield of molecular dynamics programs to define these angles. The observation of a number of dihedral angles at non-ideal staggered conformations in the disulfide bonds of our structures suggests that the internal forcefields for disulfide bonds can be better parameterized for structural characterization using NMR restraints. This is particularly problematic in CYANA where no torsion angle parameters exist for χ_2_ and χ_3_ angles, and disulfide bonds are introduced through a set of distance restraints across the disulfide bridge.

### RDCs to Define Disulfide Connectivities in Peptide Structures

Determination of disulfide-bond connectivities in DRPs remains a significant area of research without a clear and unique solution (Mobli and King, 2010; Poppe et al., 2012; Lakbub et al., 2018). An interesting approach is to measure precise distances across the disulfide bond using selective deuteration (Takeda et al., 2012). The method provides excellent accuracy of the distance between hydrogen atoms across the disulfide bond and may be used to infer disulfide-bond connectivitites (in the absence of chemical shift overlap). Similarly, disulfide proxies may be used in the form of ^77^Se enriched seleno-cystines, allowing for unequivocal determination of diselenide connectivities (Mobli et al., 2009). The methods, however, require highly specialized labeling strategies, placing them beyond routine use.

The question then remains what impact RDCs may have on resolving disulfide-bond connectivities. The above analysis of the geometry of disulfide bonds shows that although the position of the C_β_ atoms may be resolved using RDCs, it is unlikely that the RDC data will resolve the position of the sulfur atoms uniquely in solution. Further, our analysis of the quality of our structures, shows that inclusion of RDCs results in an improvement in resolution from ~2.5 Å to < 1.5 Å when RDCs are included as restraints. Based on this information, we downloaded all structures in the protein databank (PDB) that contain a disulfide bond, have a crystallographic resolution of < 1.5 Å and have a molecular weight <50 kDa (2019-09-28). We further excluded highly homologous structures (only including one representative structure when sequences have >90% identity). This resulted in a dataset of ~900 structures. We then queried C_β_–C_β_ distances between atoms in a disulfide bond (within the same chain) and also extracted C_β_–C_β_ distances for atoms that are not in a disulfide bond (regardless of chain).

Analysis of the PDB database showed that C_β_-C_β_ distances between residues in a disulfide bond (intra) overlap with those not in a disulfide bond (inter). The intra-disulfide bonds (2447 bonds in our data set) have C_β_–C_β_ distance shorter than 5 Å (average of 3.8 A ± 0.18)—note that one highly strained outlier was removed (1SO7.pdb). Further, our data contains approximately 200 C_β_–C_β_ distance shorter than 5 Å between cysteine residues not in a disulfide bonds (inter). This would suggest that finding a solution using NMR data may be difficult based on the C_β_ positions alone.

However, manual inspection of the 20 structures with the shortest C_β_–C_β_ distances of non-connected cysteines shows that such a connection would result in significant violations of other disulfide bonds. There are two particular violations that can be observed, the first is that accommodating the shorter inter-disulfide bond connection results in at least one other disulfide bond having a C_β_–C_β_ distance ≥ 5 Å. The second observation is that in all cases reviewed we find that correctly paired cysteines yield the shortest average C_β_–C_β_ distances overall. It would, therefore, seem reasonable to determine disulfide bond connectivities from such data by minimizing the C_β_–C_β_ distances between connected cysteine pairs.

Practically, this approach can be implemented by repeating the structure calculation step for each possible disulfide isoform and choosing the solution that provides the shortest overall C_β_-C_β_ distances. Historically, this approach has been applied where the structural constraints are used to optimize an appropriate function (Jordan et al., 2009). However, when only NOE data are used this approach may yield ambiguous results which has in the past led to incorrect conclusions [see discussions elsewhere (Mobli and King, 2010; Poppe et al., 2012)]. Our analysis suggests that including RDCs in such a data-driven approach provides much higher confidence in determining disulfide bond connectivities and is unlikely to lead to incorrect solutions.

## Conclusion

Structural characterization of disulfide-rich peptides is chiefly conducted using NMR spectroscopy. Although, these molecules have excellent properties for solution studies, the presence of multiple disulfide bonds poses a significant challenge in attainable resolution.

Analysis of the structure of a largely helical disulfide-rich peptide (Ta1a), using RDCs, shows that although the structure had been determined at very high precision, the overall resolution of the structure was consistent with an X-ray crystallographic resolution of ~2.5 Å. Including RDCs as restraints improves this resolution to < 1.5 Å resolution.

We find that despite inclusion of RDC restraints non-ideal geometries of cysteine bridges are found where evidence of rotamer averaging is present. We further find that χ_2_ and χ_3_ angles may display heterogeneity that cannot be resolved by RDCs alone.

Finally, we note that at the resolution achieved here, C_β_–C_β_ distance measurements are sufficient to determine disulfide-bond connectivities with high confidence.

## Data Availability Statement

The datasets generated for this study can be found in the Protein Data Bank 6URP.

## Author Contributions

MM conceived and directed the project. VR prepared all of the samples and conducted the experiments with input and guidance from all authors. VR, YS, and MM analyzed the data. VR and MM prepared the figures and tables and wrote the manuscript with input from all authors.

### Conflict of Interest

The authors declare that the research was conducted in the absence of any commercial or financial relationships that could be construed as a potential conflict of interest.
