# Supplementary material for: Residual Dipolar Couplings for Resolving Cysteine Bridges in Disulfide-Rich Peptides
Source: Front Chem. 2020 Jan 22;7:889. doi: 10.3389/fchem.2019.00889 (PMC6987419; doi:10.3389/fchem.2019.00889)
Supplement: Supplementary file 1 [file Data_Sheet_1.pdf]

Residual dipolar couplings for resolving cysteine bridges in disulfide-rich peptides

Venkatraman Ramanujam[1,2], Yang Shen[2], Jinfa Ying[2], & Mehdi Mobli[1*]

1. *Centre for Advanced Imaging, The University of Queensland, St Lucia, 4072, Queensland, Australia*
2. *Laboratory of Chemical Physics, National Institute of Diabetes and Digestive and Kidney Diseases, Bethesda, MD 20892, USA*

Correspondence: Mehdi Mobli, m.mobli@uq.edu.au

Figures

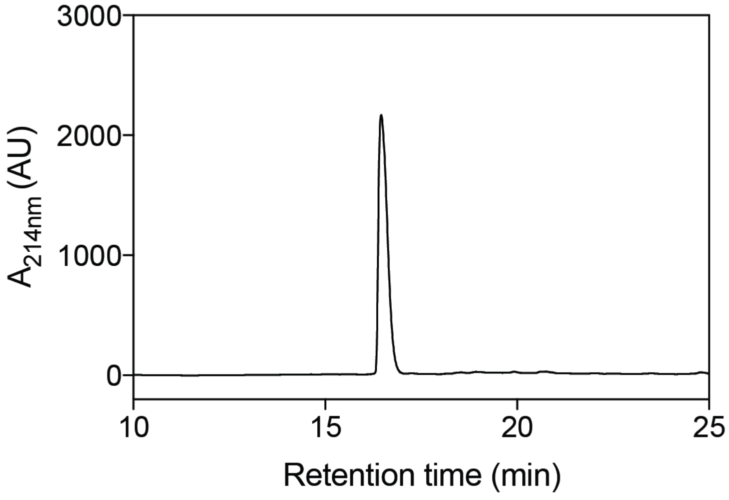

Figure S1. Purity of recombinantly produced Ta1a. Purified Ta1a elutes as single isoform at 16.4 min corresponding to 25.5% acetonitrile concentration in C3 semi-prep column.

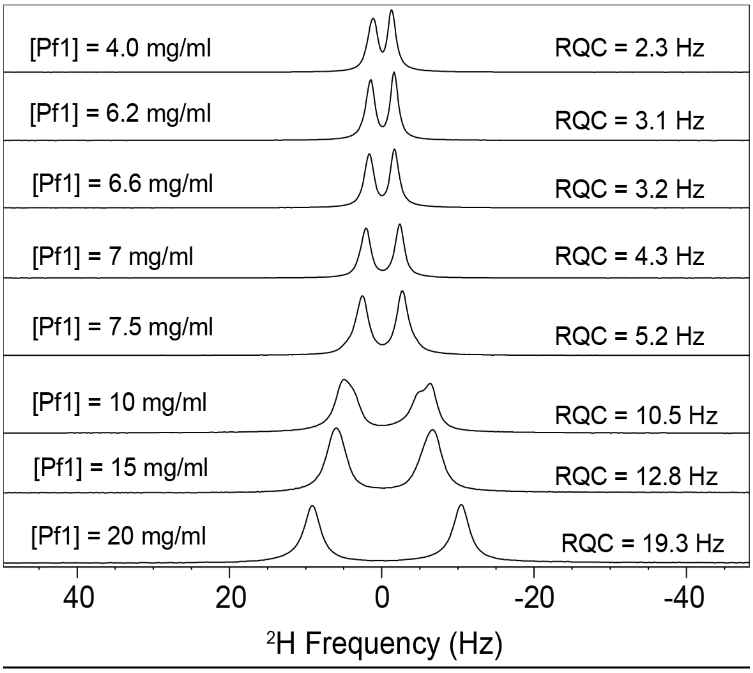

Figure S2. Residual ^{2}H quadrupolar coupling (RQC) as a function of Pf1 phage concentration in the sample containing ^{13}C/^{15}N labeled Ta1a in 20 mM phosphate buffer, 5% D_2O, pH 6.2 measured at 298K in 600 MHz. One dimensional ^{2}H spectra of Ta1a sample at various Pf1 concentrations and its corresponding ^{2}H splitting.

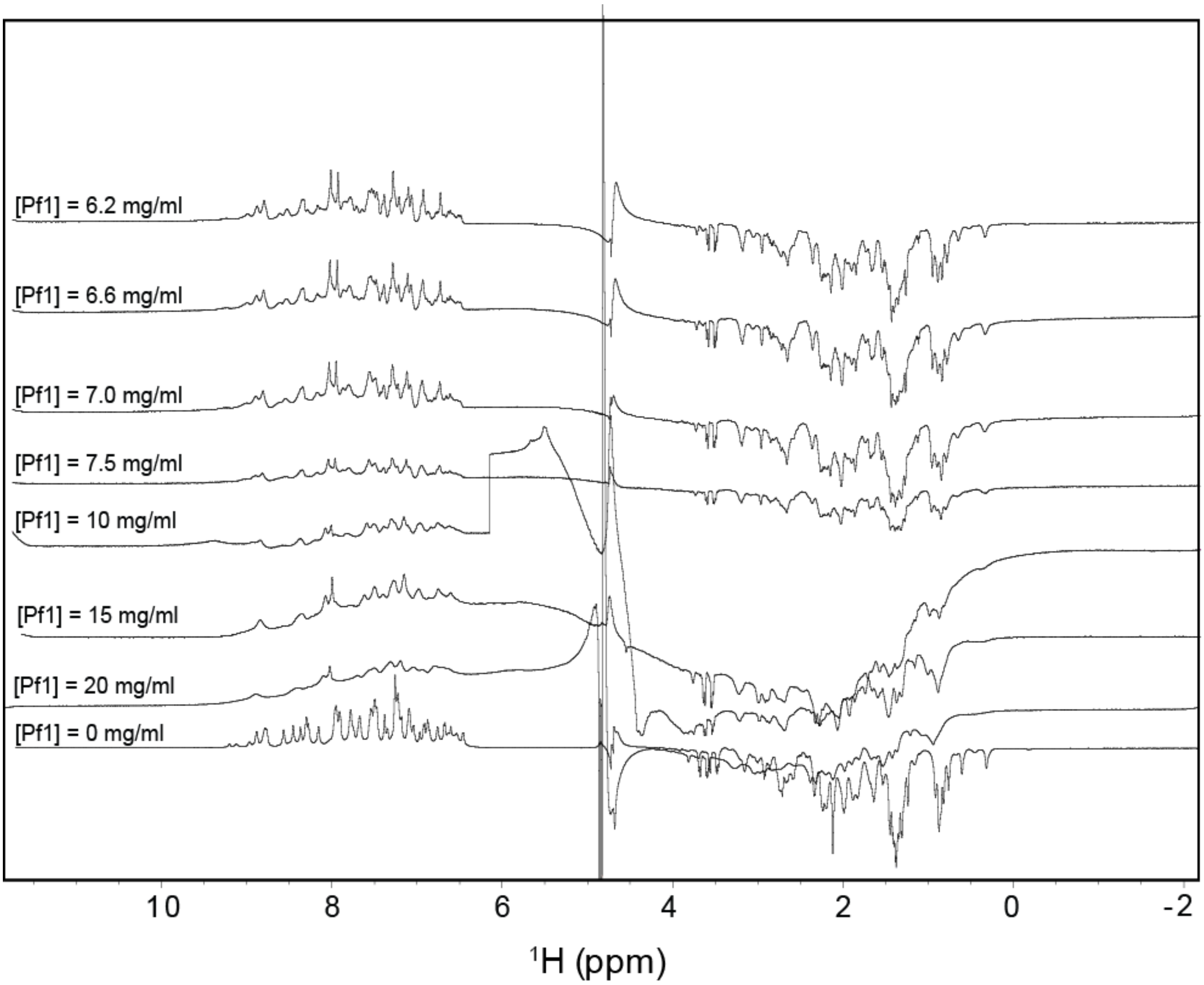

Figure S3. ^{1}H NMR spectrum, one-one echo sequence (Sklenář and Bax, 1987), of Ta1a aligned in different concentrations of Pf1 phage.

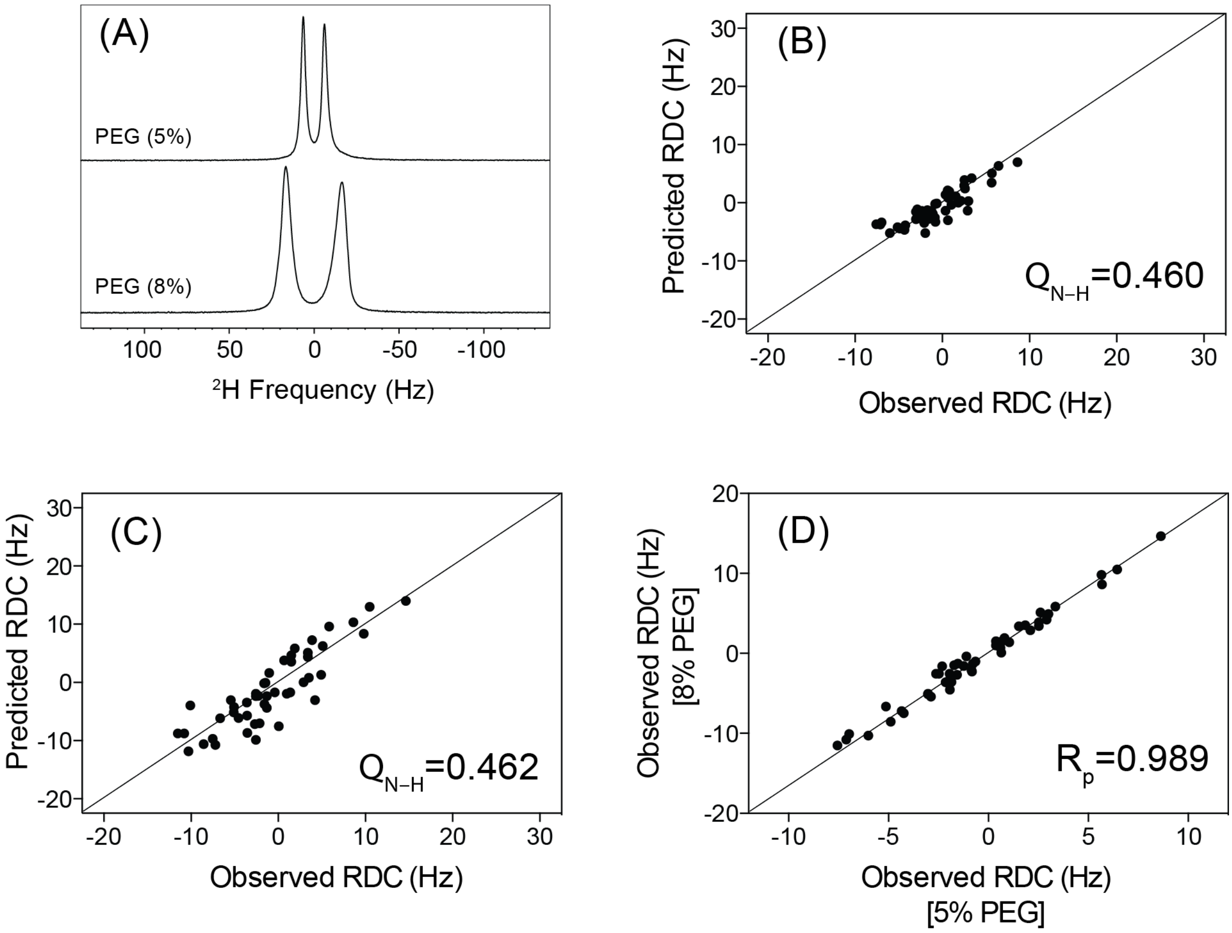

Figure S4. Optimization of PEG concentration for RDC measurements in Ta1a. (A) ^{2}H spectra of uniformly ^{15}N labeled Ta1a aligned in 5% and 8% PEG solution showing an RQC of 13.0 and 32.1 Hz respectively. (B&C) Scatter plot of correlation between experimental and structure based $^1D_{NH}$ in 5% and 8% PEG solution respectively. (D) Correlation plot of $^1D_{NH}$ observed for 5% and 8% PEG solution.

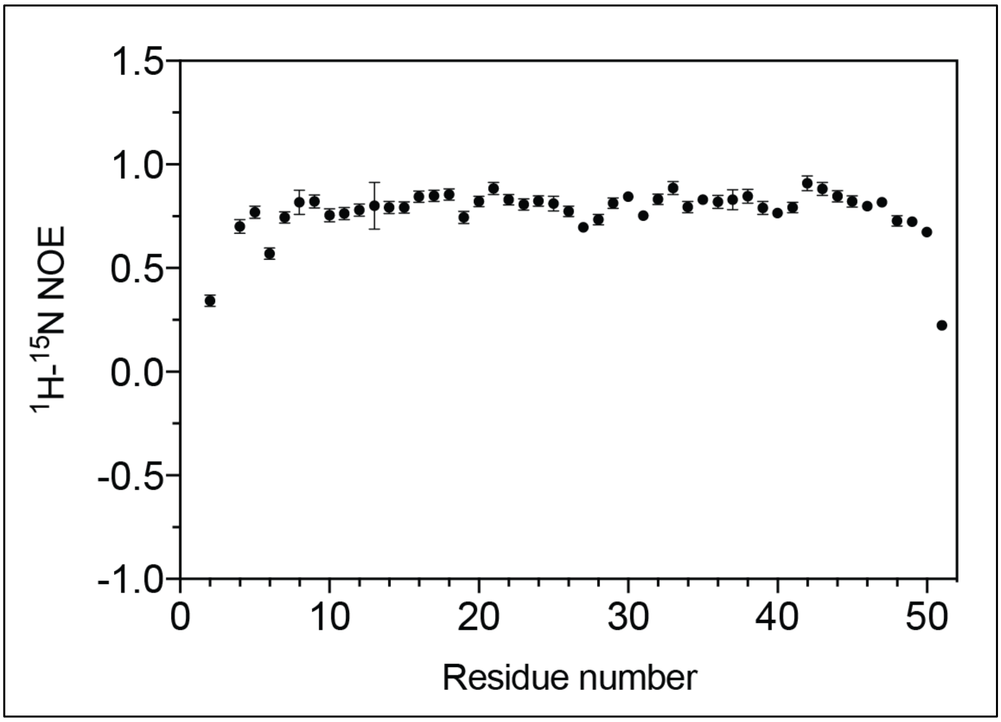

Figure S5 ^{1}H-^{15}N steady state NOE for Ta1a at a field-strength of 900 MHz. Data were acquired at 25˚C using a 200 µM sample of ^{15}N labeled Ta1a. The errors are a function of the S/N in the acquired spectra.

Tables

Table S1. Summary of NMR spectra recorded for Ta1a sample.

Expt. No	Spectra	*J*-coupling type	^{1}H frequency / MHz		
			Isotropic	Pf1	PEG
1	IPAP-HSQC	$^1J_{NH}$	900	600	600
2	HNCO	$^1J_{C\alpha C'}$	600	600	600
3	CT-HN(CO)CA	$^1J_{C\alpha H\alpha}$	600	600	600
4	^{1}H-^{15}N TROSY	$^1J_{NC'}$	600	600	600
5	CT-HN(COCA)CB	$^1J_{C\beta H\beta 2}$+$^1J_{C\beta H\beta 3}$	700	600	-
6	HA[HB,HN](CACO)NH	$^3J_{H\alpha H\beta}$	700	-	-
7	HNHB	$^3J_{NH\beta}$	900	-	-

Table S2. Experimental acquisition parameters for NMR experiments conducted. The ^{15}N carrier frequency was set 116.5 ppm, the ^{13}Cα to 56 ppm, the ^{13}C' to 177 and the Cα-Cβ to 41. Where values deviate from these, these are provided in the Materials & Methods section of the manuscript. The number of samples refers to complex points, the number of increments is half of this value in traditional sampling.

		t1	t2	t3	Scans	Inter-scan delay (s)	^{1}H Freq. (MHz)
3D CT HNCA (isotropic & anisotropic)	Nucl.	^{13}C	^{15}N	^{1}H	4	1.5	600.1
	Samples	202	110	2048			
	Acq. (ms)	26.7	39.3	122.9			
2D IPAP-HSQC - isotropic	Nucl.	^{15}N	^{1}H		8	1.5	900.1
	Samples	1600	2048				
	Acq. (ms)	336.0	94.6				
2D IPAP-HSQC - anisotropic	Nucl.	^{15}N	^{1}H		32	1.5	600.4
	Samples	1000	1600				
	Acq. (ms)	311.8	95.0				
3D CT-HN(CO)CA - isotropic	Nucl.	^{13}C	^{15}N	^{1}H	4	1.5	600.4
	Samples	200	110	2048			
	Acq. (ms)	28.0	39.3	122.9			
3D CT-HN(CO)CA – anisotropic (Pf1)	Nucl.	^{13}C	^{15}N	^{1}H	8	1.5	600.4
	Samples	166	110	2048			
	Acq. (ms)	23.2	39.3	122.9			
3D CT-HN(CO)CA – anisotropic (PEG)	Nucl.	^{13}C	^{15}N	^{1}H	8	1.5	600.4
	Samples	200	110	2048			
	Acq. (ms)	28.0	39.3	122.9			
3D CT-HNCO – anisotropic	Nucl.	^{13}C	^{15}N	^{1}H	4	1.5	600.4
	Samples	280	116	2048			
	Acq. (ms)	102.8	41.4	127.8			
3D CT-HNCO – isotropic	Nucl.	^{13}C	^{15}N	^{1}H	4	1.5	600.4
	Samples	3192 NUS		2048			
	Acq. (ms)	102.8	41.4	127.8			
3D CT-HN(COCA)CB - isotropic	Nucl.	^{13}C	^{15}N	^{1}H	2	1.35	700.2
	Samples	538	90	2048			
	Acq. (ms)	26.9	27.5	112.6			
3D CT-HN(COCA)CB – anisotropic (Pf1)	Nucl.	^{13}C	^{15}N	^{1}H	8	1.5	600.1
	Samples	534	44	2048			
	Acq. (ms)	26.7	11.3	136.5			
3D HA[HB,HN](CACO)NH	Nucl.	^{15}N	^{1}H	^{1}H	8	1.5	700.2
	Samples	56	270	2048			
	Acq. (ms)	17.1	20.3	112.6			
3D HNHB	Nucl.	^{15}N	^{1}H	^{1}H	16	1.5	900.1
	Samples	40	128	2048			
	Acq. (ms)	6.1	7.1	81.1			
2D ^{15}N-HSQC (Heteronuclear NOE)	Nucl.	^{15}N	^{1}H		32	4	900.1
	Samples	200	2048				
	Acq. (ms)	43.8	81.9				
^{13}C HSQC-NOESY	Nucl.	^{1}H	^{13}C	^{1}H	8	1	900.1
	Samples	112	56	2048			
	Acq. (ms)	5.4	1.5	75.8			
^{15}N HSQC-NOESY	Nucl.	^{15}N	^{1}H	^{1}H	16	1.5	900.1
	Samples	136	80	2048			
	Acq. (ms)	6.3	15.7	75.8			

Table S3. χ1 classification using *J*-couplings for β-methylene protons containing residues in Ta1a.

Res[#]	AA type	$^{3}J_{H\alpha H\beta 2}$ Hz	$^{3}J_{H\alpha H\beta 2}$ upl[a] Hz	$^{3}J_{H\alpha H\beta 3}$ Hz	$^{3}J_{H\alpha H\beta 3}$ upl[a] Hz	$^{3}J_{N\text{-}H\beta 2}$ Hz	$^{3}J_{N\text{-}H\beta 3}$ Hz	χ1 deg
1	SER[b]	6.96	7.04	n.d[c]	4.78	–[d]	–	average
2	GLU[b]	–	–	–	–	medium	medium	average
3	PRO[b]	6.96	6.98	6.33	6.62	–	–	average
4	ASP[b]	6.39	6.42	5.50	5.64	large	small	average
5	GLU[e]	8.64	8.64	n.c[f]	n.c	n.q[f]	n.q	n.c
7	CYS[b]	8.27	8.37	6.73	6.89	medium	medium	average
8	ARG[b]	7.93	7.99	5.63	5.89	medium	medium	average
10	ARG[b]	8.33	8.42	n.d	3.69	n.a[g]	n.a	average
11	MET[b]	5.78	5.96	n.d	3.96	medium	medium	average
13	HIS[e]	8.81	9.06	n.c	n.c	n.q	n.q	n.c
14	LYS[b]	7.22	7.39	6.49	6.51	medium	medium	average
15	GLU[b]	7.57	7.61	6.67	6.71	medium	medium	average
16	PHE	n.d	4.54	10.81	10.84	small	small	180°
17	ASN	9.18	9.21	n.c	n.c	n.q	n.q	n.c
18	TYR	n.d	4.21	10.12	10.12	small	small	180°
19	LYS	11.03	11.20	n.d	4.84	small	large	-60°
20	SER[b]	n.d	5.22	n.d	5.22	medium	medium	average
21	ASN[b]	8.09	8.25	6.44	6.50	medium	medium	average
23	CYS	11.27	11.28	n.d	4.80	small	large	-60°
24	ASN[e]	8.42	8.48	n.c	n.c	n.q	n.q	n.c
26	CYS	n.d	4.04	9.46	9.48	small	small	180°

28	ASP[b]	6.42	6.70	n.d	3.50	medium	medium	average
29	GLN	10.73	10.77	n.d	4.32	small	large	-60°
33	CYS	n.d	4.78	10.57	10.63	small	small	180°
34	GLU	9.49	9.71	4.01	4.01	small	large	-60°
36	GLU	9.23	9.31	n.d	5.30	small	large	-60°
37	CYS[b]	7.54	7.59	n.d	5.37	small	small	average
38	PHE	10.91	11.27	n.d	5.59	small	large	-60°
39	ARG[e]	8.32	8.42	n.c	n.c	n.q	n.q	n.c
40	ASN	4.52	4.80	3.48	3.70	large	small	60°
41	ASP	10.37	10.48	4.86	5.11	small	large	-60°
43	TYR	4.75	5.13	10.83	11.02	small	small	180°
46	CYS	10.31	10.32	n.d	3.83	small	large	-60°
47	HIS	5.59	5.92	10.92	11.05	small	small	180°
48	GLU[e]	9.20	9.27	n.c	n.c	n.q	n.q	n.c
50	GLN	8.69	8.75	4.18	4.21	small	large	-60°
51	LYS[b]	–	–	–	–	medium	medium	average

[a]calculated from the lowest contour level without seeing a peak; [b]stereospecific designations not meaningful; [c]not detected. i.e. below the signal-to-noise threshold; [d]couplings involving resonances non-observable; [e]residues with degenerate $H_{\beta2}/H_{\beta3}$ chemical shifts; [f]not calculated (or not qualitatively assigned) due to overlap of $H_{\beta2}$ and $H_{\beta3}$ chemical shifts; [g]not measured due to overlap of the diagonal signal.

Table S4 Classification of χ_1 torsion angles for β-methine proton containing residues in Ta1a.

Res#	AA type	$^3J_{H\alpha H\beta}$ / Hz	$^3J_{H\alpha H\beta}$ upl[a] / Hz	χ_1 /(deg)
6	ILE	8.21	8.32	average
12	THR	n.d[b]	6.22	average
22	VAL	9.72	9.80	180°
30	VAL	7.18	7.29	average
42	VAL	10.63	10.68	180°
44	THR	8.29	8.34	-60°

[a]calculated from the lowest contour level without seeing a peak; [b]not detected. i.e. below the signal-to-noise threshold.

Table S5 χ1 Classification using [1]H-^{1}H NOEs for β-methylene protons in Ta1a.

Res[#]	AA type	$d_{\alpha\beta2}$(i,i)	$d_{\alpha\beta3}$(i,i)	$d_{N\beta2}$(i,i)	$d_{N\beta3}$(i,i)	χ1 (deg)
1	SER[a]	strong	weak	–[c]	–	n.c[e]
2	GLU[a]	strong	weak	n.d[d]	n.d	n.c
3	PRO[a]	strong	weak	–	–	n.c
4	ASP[a]	strong	strong	strong	medium	average
5	GLU[b]	n.q[e]	n.q	n.q	n.q	n.c
7	CYS[a]	strong	strong	strong	strong	average
8	ARG[a]	weak	strong	medium	strong	average
10	ARG[a]	strong	weak	strong	weak	average
11	MET[a]	strong	strong	strong	weak	average
13	HIS[b]	n.q	n.q	n.d	n.d	n.c
14	LYS[a]	weak	strong	strong	strong	average
15	GLU[a]	strong	strong	strong	strong	average
16	PHE	strong	weak	strong	strong	180°
17	ASN[b]	n.q	n.q	n.q	n.q	n.c
18	TYR	strong	weak	strong	strong	180°
19	LYS	weak	strong	strong	weak	-60°
20	SER[a]	strong	weak	weak	strong	average
21	ASN[a]	strong	weak	strong	weak	average
23	CYS	weak	strong	strong	weak	-60°
24	ASN[b]	n.q	n.q	n.q	n.q	n.c
26	CYS	strong	weak	strong	medium	180°
28	ASP[a]	strong	strong	medium	strong	average
29	GLN	weak	strong	strong	medium	-60°
33	CYS	strong	weak	strong	strong	180°
34	GLU	weak	strong	strong	weak	-60°
36	GLU	weak	strong	weak	strong	average

37	CYS[a]	strong	strong	medium	strong	average
38	PHE	weak	strong	weak	strong	average
39	ARG[b]	n.q	n.q	n.q	n.q	n.c
40	ASN	strong	strong	medium	strong	60°
41	ASP	weak	strong	medium	strong	average
43	TYR	strong	weak	medium	strong	180°
46	CYS	weak	strong	strong	weak	-60°
47	HIS	strong	weak	medium	strong	180°
48	GLU[b]	n.q	n.q	n.q	n.q	n.q
50	GLN	weak	strong	strong	weak	-60°
51	LYS[a]	weak	strong	weak	strong	average

[a]stereospecific designations not meaningful; [b]residues with degenerate $H_{\beta 2}/H_{\beta 3}$ chemical shifts; [c]couplings involving resonances non-observable; [d]not detected; [e]not calculated (or peak quality not qualitatively assigned) due to peak overlap.

Table S6 Estimation of χ_1 angles from one-bond dipolar couplings. The sum of the C_β–$H_{\beta 2}$ and C_β–$H_{\beta 3}$ residual dipolar couplings (RDCs) are compared to sums of pairs of backbone RDCs. Close agreement indicates

Residue	χ_1 ~ 180° $D_{C\alpha H\alpha}+D_{(C\alpha\ C')}{}^{b}$ (Hz)	χ_1 ~ 60° $D_{C\alpha H\alpha}+D_{(C\alpha C')}{}^{a,b}$ (Hz)	χ_1 ~ -60° $D_{(C\alpha C')}{}^{b}+D_{(C\alpha C')}{}^{a,b}$ (Hz)	**$\Sigma D_{C\beta H\beta}$ (Hz)**	*$\chi_1{}^{c}$ (deg)*
Phe-16	3.35	49.50	30.38	**-2.40**	*180°*
Cys-26	13.92	-9.83	77.46	**26.75**	*180°*
Asn-40	-59.52	26.65	18.77	**26.15**	*60°*
Tyr-43	-56.73	1.96	-3.73	**-58.69**	*180°*
His-47	-54.36	14.81	-3.63	**-56.60**	*180°*

[a]obtained from the $D_{C'C\alpha}$ of the preceding residue; [b]values normalized to $D_{C\alpha H\alpha}$ by multiplication by 10.37; [c]estimated by comparing the $\Sigma D_{C\beta H\beta}$ to backbone $D_{C'C\alpha}$ and $D_{C\alpha H\alpha}$.

Table S7. Structural statistics for ensembles of Ta1a

	Original structure	Refined structure
Experimental restraints		
Distance restraints (CYANA)		
Short-range (i-j<=1)	349	350
Medium-range (1<i-j<5)	137	143
Long-range (i-j>=5)	88	85
Dihedral angle restraints	93	99
Disulfide-bond restraints	9	9
RDC restraints		
$^{1}D_{NH}$	-	96
$^{1}D_{C\alpha H\alpha}$	-	96
$^{1}D_{NC}$	-	88
$^{1}D_{C\alpha C'}$	-	95
$^{1}D_{(C\beta H\beta 2+C\beta H\beta 3)}$	-	37
Total number of restraints per residue	13.3	21.7
Q-factor^{Pf1}	0.39	0.08
Q-factor^{PEG}	0.58	0.21
RMSD from mean structure in Å		
(residues from 4 helices used and given in brackets)		
Backbone atoms (5-10, 13-24, 30-35, 41-49)	0.41	0.24
All heavy atoms (5-10, 13-24, 30-35, 41-49)	1.71	1.63

Table S8 Classification of disulfide bonds in conformational categories (Schmidt et al., 2006) from NOE-derived structure of Ta1a.

Ensemble structures[a]	Disulfide conformation[b]		
	Cys_7-Cys_{37}	Cys_{23}-Cys_{33}	Cys_{26}-Cys_{46}
Model 1	*-RHHook*	*-LHSpiral*	*+/-RHSpiral*
Model 2	*-RHHook*	*-LHSpiral*	*+/-RHSpiral*
Model 3	*-RHHook*	*+/-LHSpiral*	*+/-RHSpiral*
Model 4	*-RHStaple*	*+/-LHSpiral*	*+/-RHSpiral*
Model 5	*-LHSpiral*	*+/-LHSpiral*	*+/-RHSpiral*
Model 6	*-RHStaple*	*-LHSpiral*	*+/-RHSpiral*
Model 7	*-LHSpiral*	*-LHSpiral*	*+/-RHSpiral*
Model 8	*-RHStaple*	*+/-LHSpiral*	*+/-RHSpiral*
Model 9	*-RHHook*	*-LHSpiral*	*+/-RHSpiral*
Model 10	*-RHStaple*	*-LHSpiral*	*+/-RHSpiral*
Model 11	*-RHHook*	*+/-LHSpiral*	*+/-RHSpiral*
Model 12	*-RHStaple*	*-LHSpiral*	*+/-RHSpiral*
Model 13	*-RHHook*	*+/-LHSpiral*	*+/-RHSpiral*
Model 14	*-LHSpiral*	*+/-LHSpiral*	*+/-RHSpiral*
Model 15	*-LHSpiral*	*-LHSpiral*	*+/-RHSpiral*
Model 16	*-RHHook*	*-LHSpiral*	*+/-RHSpiral*
Model 17	*-RHHook*	*-LHSpiral*	*+/-RHSpiral*
Model 18	*-RHHook*	*-LHSpiral*	*+/-RHSpiral*
Model 19	*-LHSpiral*	*+/-LHSpiral*	*+/-RHSpiral*
Model 20	*-RHStaple*	*-LHSpiral*	*+/-RHSpiral*
Model 21	*-LHSpiral*	*+/-LHSpiral*	*+/-RHSpiral*
Model 22	*-LHSpiral*	*-LHSpiral*	*+/-RHSpiral*
Model 23	*-RHHook*	*-LHSpiral*	*+/-RHSpiral*
Model 24	*-RHHook*	*-LHSpiral*	*+/-RHSpiral*

Model 25	*-RHStaple*	*-LHSpiral*	*+/-RHSpiral*

[a]NOE-derived structure (PDB: 2KSL); [b]LH:Left handed oriented; RH:Right handed oriented; -:Negative value for the respective dihedral angle; +:Positive value for the respective dihedral angle.

Reference

Schmidt, B., Ho, L., and Hogg, P.J. (2006). Allosteric disulfide bonds. *Biochemistry* 45(24)**,** 7429-7433. doi: 10.1021/bi0603064.

Sklenář, V., and Bax, A. (1987). Spin-echo water suppression for the generation of pure-phase two-dimensional NMR spectra. *J Magn Reson (1969)* 74(3)**,** 469-479. doi: 10.1016/0022-2364(87)90269-1.
